# The Toxicological Risk Assessment (TRA) of Total Chromium Impurities in *Menthae piperitae tinctura* (*Mentha x piperita* L., folium) Available in Polish Pharmacies Including Regulatory Approaches with Special Emphasis of Cr Speciation and Genotoxicity

**DOI:** 10.1007/s12011-022-03367-4

**Published:** 2022-07-28

**Authors:** Kamil Jurowski, Mirosław Krośniak

**Affiliations:** 1 Institute of Medical Studies, Medical College, Rzeszów University, Al. mjr. W. Kopisto 2a, 35-959 Rzeszów, Poland; 2 Department of Food Chemistry and Nutrition, Medical College, Jagiellonian University, Medyczna 9, 30-688 Kraków, Poland

**Keywords:** Chromium impurities, Menthae piperitae tincture, Toxicological risk assessment of impurities, Margin of exposure, Permitted daily exposure, Toxicity of chromium

## Abstract

Chromium is pharmacologically active and is not an essential element but is still very intriguing and demanding from the point of view of toxicological risk assessment. Especially as an elemental impurity in final pharmaceuticals. The aim of this article is toxicological risk assessment (TRA) of total Cr impurities in *Menthae piperitae tinctura* (*Mentha x piperita* L., folium) available in Polish pharmacies including triple approach. Obtained raw/basal results shows that impurities of total Cr impurities were present in all investigated pharmaceutical with *Mentha x piperita* L., folium. but at a relatively low level (in the range: 0.39–2.14 µg/L). The regulatory strategy based on the ICH Q3D (R1) elemental impurities guidelines confirms that all the requirements of the analyzed products meet the European Medicine Agency (EMA) requirements. Obtained results obtained show that the estimated maximum daily exposure to Cr (ng/day) are variable between the samples (0.521–3.792 ng/day), but at a relatively low level (< 4.0 ng/day).The final step confirms the safety of analyzed pharmaceuticals, because the comparison of the estimated results with the oral PDE value for Cr in final drugs suggested by the ICH Q3D guideline (10,700 µg/day) show that all the products are below this value. The approach based on the margin of exposure (MoE) for children and adults also confirms the safety of all the products with *Mentha x piperita* L., (in all cases MoE >  > 10 000). It can be concluded that all the samples analyzed should not represent any health hazard to patients due to Cr impurities. To our knowledge, this is the first study about application of comprehensive TRA of total Cr impurities in phytopharmaceuticals with *Mentha x piperita* L., folium available in European pharmacies. Additionally, we confirm the safety of Cr impurities by applying triple regulatory strategy without the application of an expansive and demanding HPLC-ICP-MS technique for Cr speciation.

**Supplementary Information:**

The online version contains supplementary material available at 10.1007/s12011-022-03367-4.

## Introduction

**Fig. 1:**
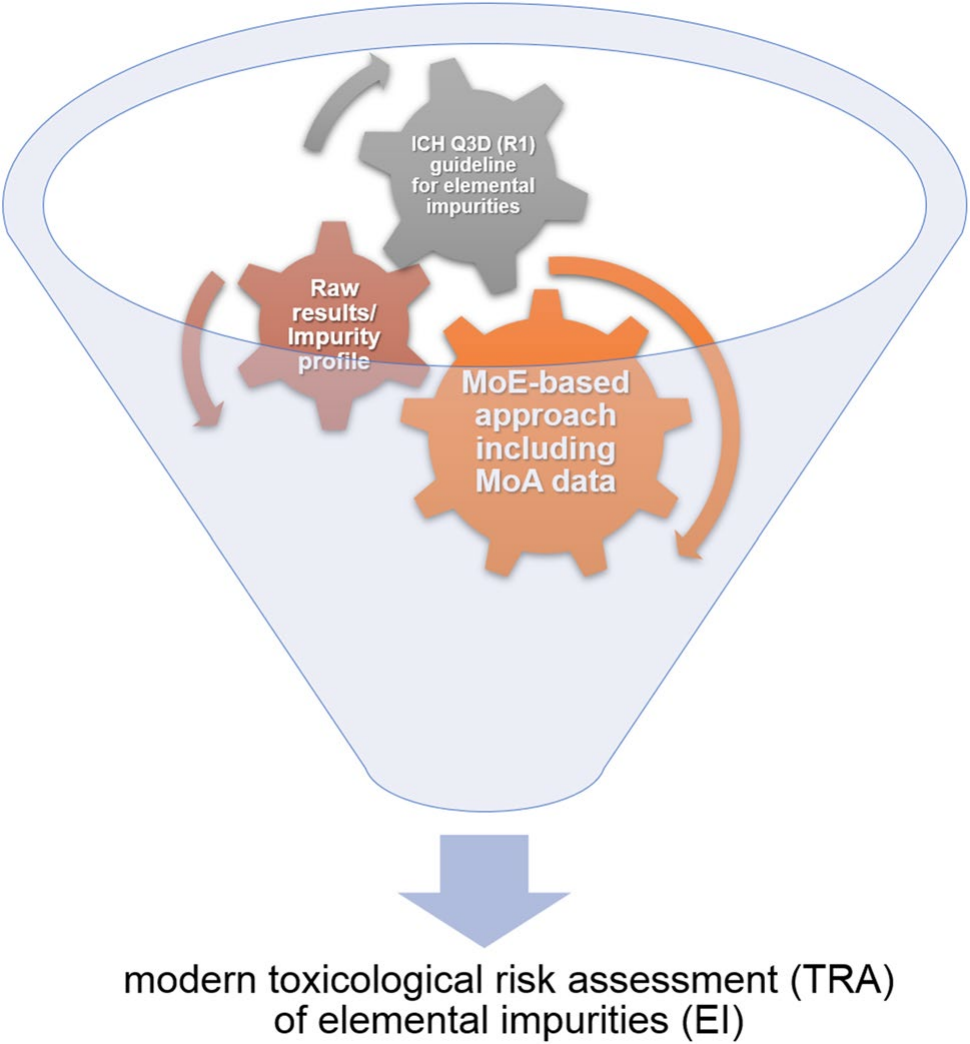
The possible approaches in of modern toxicological risk assessment (TRA) of elemental impurities in final pharmaceuticals

Studies of elemental impurities (EI) in finished pharmaceutical products/drugs are extremely rare, are treated marginally, and often refer to only routine analytical protocols for quality control for pharmaceutical industry purposes. There are many different interesting strategies for toxicological health risk assessment in the literature [1, 2], but they usually concern the analysis of food, not final pharmaceuticals. However, modern toxicological risk assessment (TRA) of final pharmaceuticals should change the classical regulatory toxicology point of view of regulatory toxicology (usually based on raw results) to more comprehensive approaches, including especially specific mode(s) of action (MoA) [3]. This type of research is currently desirable and recommended by various toxicological associations. Hence, for studies about EI in final pharmaceuticals, there is a need for extension of applied strategies because usually this kind of study is associated in most cases with the comparison of the obtained raw results with reference data. For this purpose, there are several options which are schematically summarized in Fig. 1.

Figure 1 illustrated diagram schematically illustrates the important modern relationship between the regulatory approaches presented as “cogs” of the TRA of EI in final pharmaceuticals. This comprehensive idea seems to be uncomplicated at first glance, but what if the element shows speciation phenomena? This is not only a challenge, but the need to develop appropriate strategies.

A review of the current literature shows that it is a very rare topic [4, 5]. Studies including EI in herbal medicinal products available in pharmacies are dynamically developed by the group conducted by Jurowski et al. [6–9]. Based on the literature review, elements that are characterized by the phenomenon of speciation are a major challenge. An elegant example can be chromium which, among the wide range of oxidation states, is the most important are few forms: Cr (0) (in elemental form) Cr(II), Cr(III), and Cr(VI) [10]. An additional challenge will be to determine total chromium due to the technical possibilities (appropriate analytical technique: HPLC-ICP-MS is an expensive, demanding technique, and beyond our reach). Hence, is it possible to make such a comprehensive TRA under such conditions? The case study will be total chromium impurities in herbal medicinal products (HMP) such as *Menthae piperitae tinctura* (*Mentha x piperita* L., folium) collected from Poland’s pharmacies. The justifications for conducting these studies are given below.

- This kind of HMP is registered in the EU and is very popular among European population [11].
- Chromium currently can only be considered pharmacologically active and not an essential element [12, 13].
- Speciation phenomena is very important for assessment of chromium (especially Cr(III) and Cr(VI), which is related to the specific MoA(s) important for regulatory purposes.

**Fig. 2:**
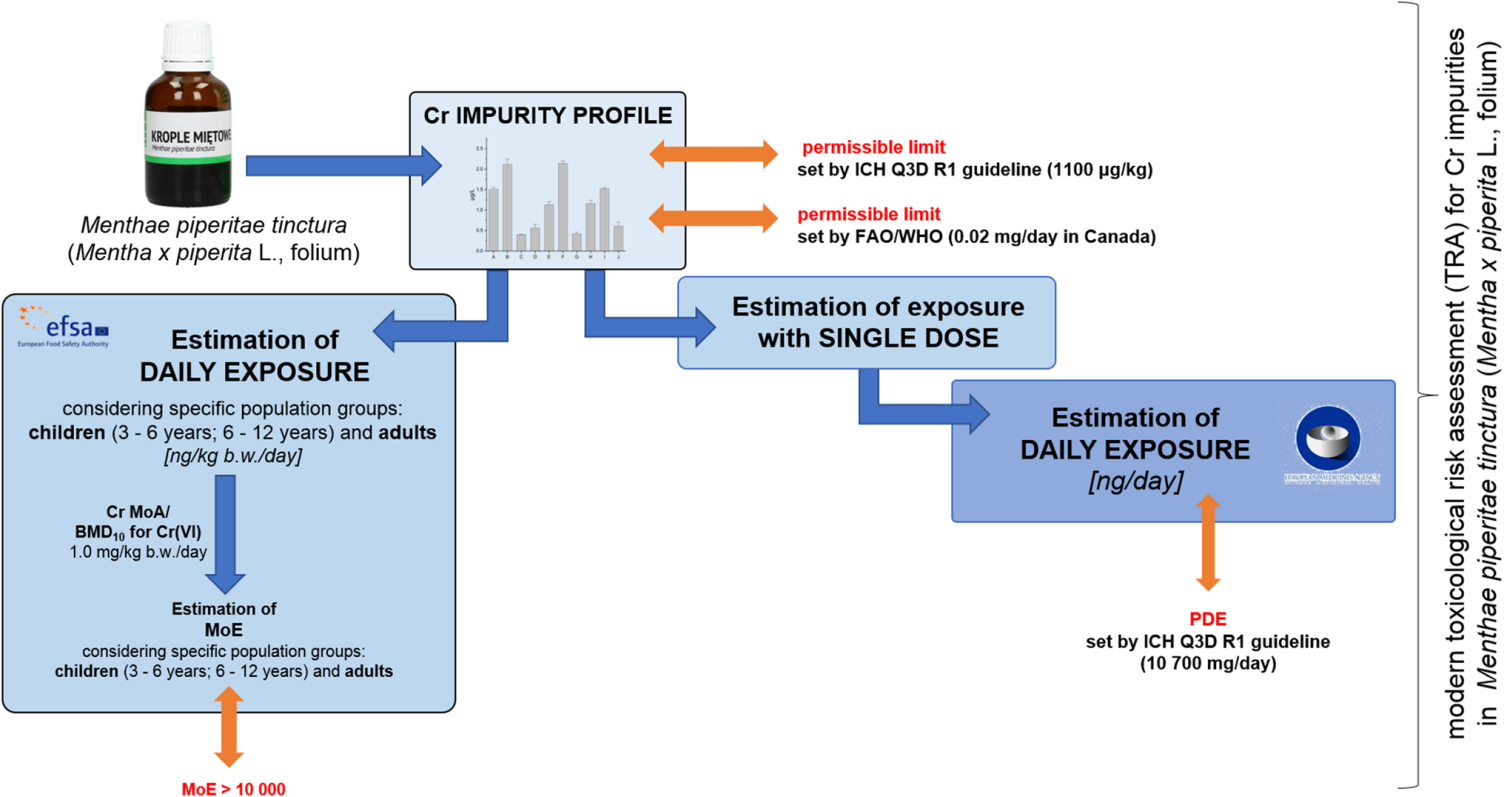
The level scheme of applied approaches in comprehensive TRA related to Cr impurities in *Menthae piperitae tinctura* (*Mentha x piperita* L., folium) collected from pharmacies in Poland

It should be emphasized that the aim of the study was not to determine total Cr impurities in HMP as tinctures with *Mentha x piperita* L., folium. The focus was not on innovations in determination, but the aim of this article was the application of modern TRA approaches appropriate to Cr impurities in *Menthae piperitae tinctura* (*Mentha x piperita* L., folium) collected from pharmacies in Poland. It should be underlined that considering the speciation of Cr, be applied should usually studies with the HPLC-ICP-MS technique, but despite the lack of access to this expensive and demanding technique, it is possible to confirm safety in terms of total Cr impurities by assuming a worst-case scenario for Cr(VI) impurities applying a MoE-based strategy for regulatory purposes. The mail idea of our work is schematically described as the level scheme in Fig. 2.

## Materials and Methods

### Samples and Chemicals

In our studies, we analyzed HMP registered in EU as *Menthae piperitae tinctura* collected from pharmacies in Poland (Rzeszów, Kraków and Niepołomice) in period: January 2022 to March 2022. It should be emphasized that we have analyzed all available HMP with *Mentha x piperita* L., folium in Poland (*n* = 10). It should be noted that only a few independent manufacturers produce these types of pharmaceutical products in Poland, hence the relatively low number of available samples. All products considered belong to over-the-counter medications. To maintain the highest methodological standards, we coded all samples as (A–J). The description of all the samples analyzed is summarized in Table 1. It should be noted that the pretreatment and treatment steps of the samples (homogenization and digestion in nitric acid) were unnecessary because all samples were in liquid form (drops). Therefore, in situ analysis was applied at the measurement step.Applied solutions were prepared using ultrapure demineralised water obtained from the Milli-Q water purification system (Millipore, Bedford, MA, USA). For calibration, working solutions of Cr (0.0, 12.5, 20.0, 50.0, and 100.0 μg/L) were prepared from the stock solutions of 1 mg/mL chromium(III) nitrate (CertiPUR®) applying demineralised water (mentioned earlier) in 0.5 mol/L HNO_3_. The certified reference material (BCR-482; IRMM, Belgium) was material prepared from lichen. The purge gas was argon at 5 N purity.

**Table 1:** Summary of investigated HMP with *Mentha x piperita* L., folium

Code	Description	Density, g/mL	Doses			OTC
			**Children** **(8–12 y/o)**	**Adolescents** **(8–12 y/o)**	**Adults**	
A	Tincture 1:20 *Mentha x piperita* L. folium, extraction solvent ethanol 80–85% V/V	0.93	Orally: 8–10 drops/2 times daily	Orally: 10–15 drops/3 times daily	Orally: 15–20 drops/3 times daily	Yes
B	Tincture 1:20 *Mentha x piperita* L. folium, extraction solvent ethanol 90% V/V	0.92	Orally: 8–10 drops/3 times daily	Orally: 10–20 drops/2 times daily	Orally: 20–50 drops/2 times daily	Yes
C	Tincture 1:20 *Mentha x piperita* L. folium, extraction solvent ethanol 90% V/V	0.93	Orally: 8–10 drops/3 times daily	Orally: 10–15 drops/ 3 times daily	Orally: 15–25 drops/3 times daily	Yes
D	Tincture 1:20 *Mentha x piperita* L. folium, extraction solvent ethanol 90% V/V	0.90	Orally: 8–10 drops/2 times daily	Orally: 15–20 drops/ 2 times daily	Orally: 20–40 drops/2 times daily	Yes
E	Tincture 1:20 *Mentha x piperita* L. folium, extraction solvent ethanol 80–85% V/V	0.91	Orally: 10 drops/2 times daily	Orally: 10–20 drops/2 times daily	Orally: 20–35 drops/2 times daily	Yes
F	Tincture 1:20 *Mentha x piperita* L. folium, extraction solvent ethanol 90% V/V	0.92	Orally: 8–10 drops/3 times daily	Orally: 10–20 drops/2 times daily	Orally: 20–50 drops/2 times daily	Yes
G	Tincture 1:20 *Mentha x piperita* L. folium, hextraction solvent ethanol 90% V/V	0.93	Orally: 8–10 drops/3 times daily	Orally: 10–15 drops/3 times daily	Orally: 15–25 drops/3 times daily	Yes
H	Tincture 1:20 *Mentha x piperita* L. folium, extraction solvent ethanol 80–85% V/V	0.91	Orally: 10 drops/2 times daily	Orally: 10–20 drops/2 times daily	Orally: 20–35 drops/2 times daily	Yes
I	Tincture 1:20 *Mentha x piperita* L. folium, extraction solvent ethanol 80–85% V/V	0.93	Orally: 8–10 drops/2 times daily	Orally: 10–15 drops/3 times daily	Orally: 15–20 drops/3 times daily	Yes
J	Tincture 1:20 *Mentha x piperita* L. folium, extraction solvent ethanol 90% V/V	0.92	Orally: 8–10 drops/2 times daily	Orally: 15–20 drops/2 times daily	Orally: 20–40 drops/2 times daily	Yes

### Analytical Studies

Quantitative analysis of total chromium in the investigated HMP with *Mentha x piperita* L., folium was performed applying PerkinElmer 5100 ZL atomic absorption spectrometer (PerkinElmer, Norwalk, CT, USA) with Zeeman background correction and with electrothermal atomization (ET-AAS technique). All details of the applied methodology are briefly described in Supplementary Materials 1 (SM1).

### Modern Toxicological Risk Assessment (TRA) of Total Chromium Impurities in Menthae piperitae tinctura (Mentha x piperita L., Folium) Available in Polish Pharmacies

As mentioned in introduction, comprehensive risk assessment for modern regulatory purposes requires a proper strategy summarized in Fig. 2.

The first step is the preparation and analysis of the raw results (impurity profile). For this purpose, appropriate plots should be prepared for the total Cr content in all investigated samples. The next step is comparison of obtained results with permissible limits set by appropriate institutions (e.g., FAO/WHO and ICH Q3D R1 guideline).

The next step should be an approach based on EMA requirements, that is, estimation of single dose exposure (Fig. 2 on the right) and daily dose exposure for each element in all samples, and then comparison of obtained results (estimated values of daily exposure) with the corresponding permitted daily exposure (PDE) recommended by ICH Q3D R1 guideline [10]. However, considering fact that investigated samples are also applied in treatment of children and adolescents (based on posology from each manufacturer—see Table 1), an additional approach is required to cover specific population groups (MoE-based, Fig. 2 on the left).

**Fig. 3:**
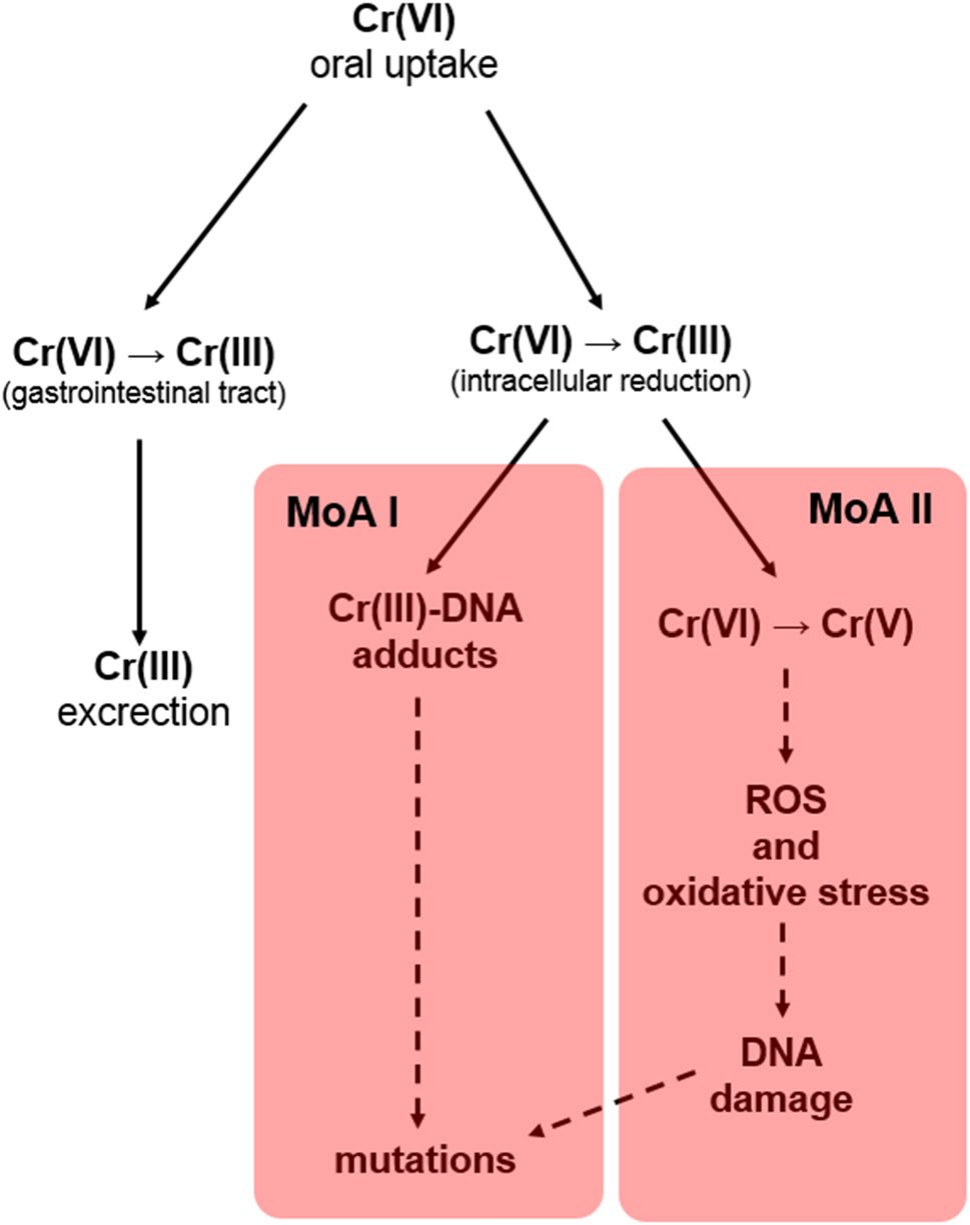
Potential modes of action (MoA) for Cr(VI) impurities after oral uptake

Hence, the last step in TRA (covering children and adolescents) should be approach based on EFSA strategy, i.e., the margin of esposure (MoE) strategy [14]. MoE can be defined as the relationship between a point of departure (POD_sys_; usually historical NOAEL or BMDL_10_ values from oral studies) and an estimate of the estimated exposure—Eq. 1.:

1 $$\mathrm{MoE }=\mathrm{ PODsys}/\mathrm{EE}$$

where:PODsys – point of departure (e.g.: mg/kg bw/day).EE – estimated exposure (e.g.: mg/kg bw/day).The MoE concept is a very useful strategy that can be applied to impurities that are both genotoxic and carcinogenic, irrespective of their origin [14]. It has been assumed that the MoE value of 10,000 (or higher) is considered of low concern from a TRA point of view with respect to the carcinogenic effect [14]. Justifications for using this approach were (1) documented in scientific literature application of this concept for different herbal products and (2) the possibility of applying to children and adolescents as a specific group of the toxicologically relevant population in our case.What about appropriate PoD_sys_? Making safety decisions that include the mode of action (MoA) of impurities should be included in modern TRA [15]. In this case, among the wide range of oxidation states, the most important are a few forms: Cr(0) (as elemental form) Cr(II), Cr(III), and Cr(VI) [2]. There is no doubt that the Cr(VI) form is the most crucial from a toxicological point of view. Taking into account the speciation phenomena according to chromium impurities, the key determinant (first MoA) for the genotoxic action of Cr(VI) (Fig. 3) is intracellular reduction *through Cr (VI)* to Cr(III). The reduction of Cr(VI) to Cr(III) is also important in an earlier phase of MoA since it is an important factor in the bioavailability of Cr(VI) upon oral intake, especially given the fact that the bioavailability of Cr(III) may be more limited than that of Cr(VI) since Cr(III) cannot easily pass cell membranes and enter cells [16]. It should be emphasized that once absorbed, Cr(VI) is reduced to Cr(III) with the formation of Cr-DNA adducts and other DNA damage resulting in mutagenesis [9, 10], which is considered the main MoA (mode of action I in Fig. 3). The second MoA is the reduction of Cr(VI) resulting in the production of Cr(V) that can result in the formation of ROS after reaction under H_2_O_2_ to generate hydroxyl radicals, ROS, and oxidative stress [17, 18], resulting in damage to DNA, and mutation (mode of action II; Fig. 3). It should be highlighted that both MoA can occur and contribute to the genotoxic effects of Cr(VI) [19].

Hence, to make safety decisions that include the MoA of chromium impurities (neoplastic effects of Cr(VI), given that BMDL_10_ of 1.0 mg Cr(VI)/kg b.w./day for the combined incidence of adenomas and carcinomas in the mouse small intestine as point of departure (PoD) [13]), the margin of exposure (MoE) approach should be applied for a comprehensive toxicological risk assessment.

## Results and Discussion

For clarity, the results and the discussion section are presented in steps summarized in Fig. 2. and described above.

### Step 1. The Impurity Profile of the Total Cr Content in the Analyzed HMP with Mentha x piperita L., Folium (A–J)

**Fig. 4:**
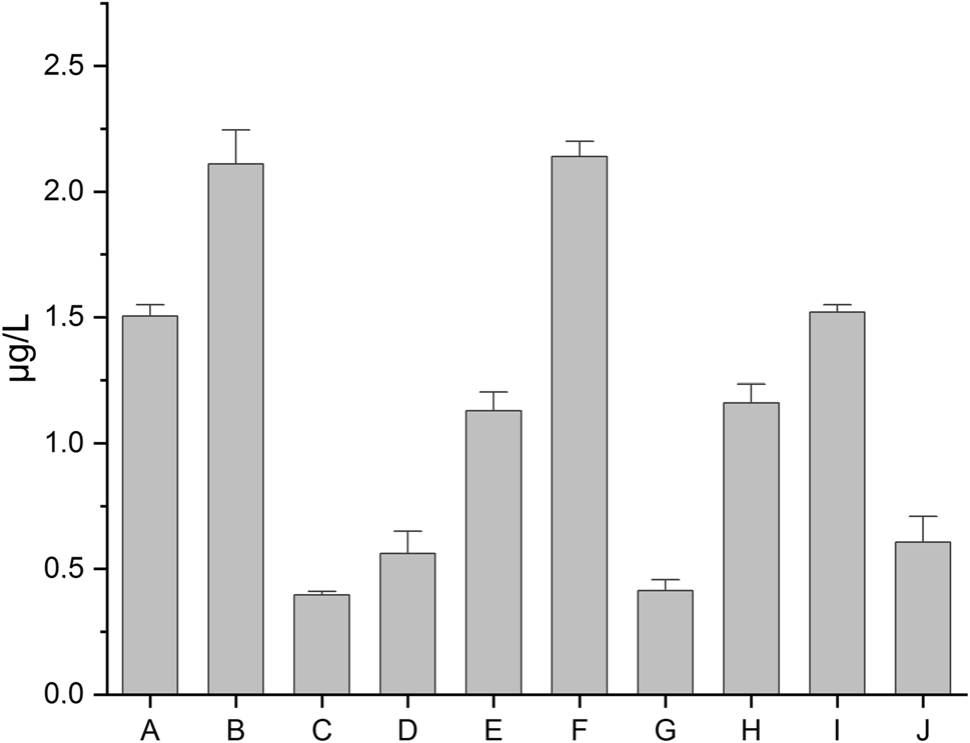
The impurity profile of total Cr content in analyzed HMP with *Mentha x piperita* L., folium (A–J)

The first step is based on raw/base results obtained from the determination of the total Cr content in all samples (A–J) which is presented in Fig. 4 (µg/L) as impurity profile.

**Fig. 5:**
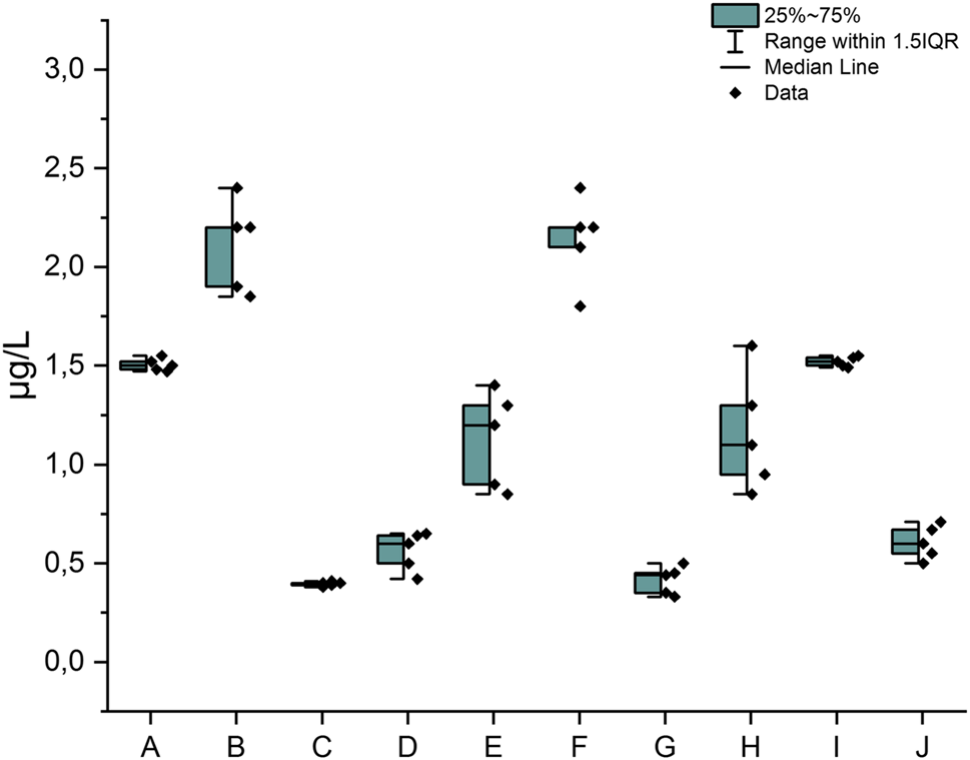
The half box for total Cr content in analyzed HMP with *Mentha x piperita* L., folium (A–J)

Additionally, the half box for total Cr content is presented in Fig. 5.

In general, Cd impurities were present in all investigated samples (raw data in Supplementary Materials 2, SM2); in the range: 0.39–2.14 µg/L. The highest and similar levels of Cr were observed in the samples: B (2.11 ± 0.08 µg/L) and F (2.14 ± 0.03 µg/L). The lowest level was observed in sample C (0.39 ± 0.03 µg/L) and also in sample G (0.41 ± 0.07 µg/L). The descriptive statistics are presented in Supplementary Materials 2 (SM2), the values of skewness (1.48; measure of asymmetry or distortion of symmetric distribution) and kurtosis (2.63; a measure of the combined weight of a distribution’s tails relative to the center of the distribution), confirm the distribution of results and their consistency.

At this stage, it is impossible to compare the results obtained with other studies because there is a lack of these kinds of studies due to the final pharmaceuticals. However, it is possible to compare raw results with permissible limits set by appropriate institutions, e.g., FAO/WHO and the ICH Q3D R1 guideline. The obtained raw results show that all samples contained Cr levels below the permissible limit set by FAO/WHO for herbal medicines (0.02 mg/day in Canada [20]). Furthermore, all results are below the level limits in final pharmaceutical products via the oral route recommended by the ICH Q3D guideline (1100 μg/kg [10]). The results obtained are baseline results, which are a starting point for further risk assessment estimations. The value of these results is not very crucial but may be valuable for other researchers for comparative purposes and may be also a reference point for regulatory purposes (data statistics for quality control protocols by institutions controlling the quality and safety of finished pharmaceutical products, e.g. National Medicines Institute in Poland).

### Step 2. The Approach Based on EMA Requirements (ICH Q3D R1 Guideline)

The second is crucial for estimating the Cr content in a a single dose and in daily oral dose. This step is based on EMA requirements (ICH Q3D R1 guideline), i.e., the estimation of total chromium content in single oral dose. For this purpose, the worst-case scenario (WCS) should be applied based on the doses of each analyzed product described by each manufacturer (see Table 1). In this approach, we assume the highest amount (drops) of orally administered tincture in a single dose (based on Table 1) and also the results from first step (Fig. 1). The estimation of the total Cr content in a single oral dose to which the patient is exposed is shown in Table 2. This step is necessary for the final step of the assessment of Cr exposure in the daily intake of investigated HMP.Then, it is possible to estimate the exposure to daily intake (ng / day), which was possible based on the frequency of use (last column in doses in Table 1). The results obtained (Table 2) show that the estimated maximum daily exposure of Cr is variable between the analyzed samples (0.521–3.792 ng/day), but at a relatively low level (< 4.0 ng/day). How to analyze the results obtained is crucial for the appropriate toxicological evaluation and usually is discussed marginally. The idea is to apply an appropriate point of departure for comparison of the obtained results with existing data. The most useful data for the evaluation of Cr impurities in pharmaceuticals were obtained from the National Toxicology Program studies [21] on the carcinogenicity of Cr(III) picolinate administered in food to rats and mice at 2000, 10,000, and 50,000 ppm. The value obtained from NOAEL was the low dose of 90 mg/kg Cr (III) picolinate (10.7 mg/kg/day Cr(III)) in rats based on the increase in the incidence of preputial gland adenoma in male rats at 460 mg/kg [10]. Therefore, the PDE value of Cr was established as 10 700 mg/day [10]. Our results for a daily dose show that all investigated HMP with *Mentha x piperita* L., folium were characterized by results extremely below the established PDE value.

**Table 2:** The estimation of Cr content in single oral dose and daily oral dose in analyzed HMP with *Mentha x piperita* L., folium (A–J)

Sample	The estimation of Cr content in a single oral dose		The estimation of Cr content in daily oral dose	
	ng/single dose	SD	ng/day	SD
A	0.527	0.08	1.582	0.09
B	1.869	0.15	3.738	0.21
C	0.174	0.04	0.521	0.08
D	0.407	0.07	0.814	0.04
E	0.708	0.09	1.417	0.10
F	1.896	0.21	3.792	0.24
G	0.192	0.05	0.576	0.09
H	0.727	0.11	1.454	0.08
I	0.533	0.08	1.598	0.06
J	0.439	0.06	0.878	0.05

*SD*, standard deviation.

### Step 3. The Approach Based on Margin of Exposure (MoE)

The last step is the approach based on MoE. As mentioned earlier, MoE is very universal and powerful “toxicological tool” that can be applied to impurities that are both genotoxic and carcinogenic, regardless of their origin. Hence, MoE-based approach is appropriate for our case study with Cr impurities. First, the values of daily exposure to a product (ng/kg bw/day) were estimated based on the amount applied and the frequency of application and the average weight of the specific population groups: children (3–6 years old; 6–12 years old) and adults. To do this, the estimation of Cr in daily dose was carried out, depending on age and body weight for each population group (based on WHO growth standards [22]) was carry out. The results obtained from a daily dose of Cr depending on specific population groups in the analyzed samples (ng/kg bw/day) are given in Table 3.The calculated values of MoE (based on Eq. 1.) for Cr in daily dose for each HMP with *Mentha x piperita* L., folium (A–J), depending on age and body weight for each specific population group are summarized in Table 4.Despite conservative assumptions, the MoE values obtained for Cr in daily dose for each HMP with *Mentha x piperita* L., folium (A–J) are greater than 10,000; therefore, exposure to Cr would not cause a health risk based on the MoE-based strategy.

**Table 3:** The estimated of Cr in daily dose for each HMP with *Mentha x piperita* L., folium (A–J), depending on age and body weight for each specific population group (ng/kg bw/day)

Specific population groups. Age	Approximatebody weight (kg)	HMP with *Mentha x piperita* L.. folium									
		A	B	C	D	E	F	G	H	I	J
Children 3–6 y/o	15–23	0.035–0.023	0.049–0.032	0.009–0.006	0.013–0.009	0.026–0.017	0.050–0.033	0.010–0.006	0.027–0.018	0.036–0.023	0.014–0.009
Children 6–12 y/o	23– 46	0.052–0.026	0.072–0.036	0.014–0.007	0.019–0.010	0.039–0.019	0.073–0.037	0.014–0.007	0.040–0.020	0.052–0.026	0.021–0.010
Adults	60	0.026	0.037	0.007	0.010	0.020	0.038	0.007	0.020	0.027	0.011

*y/o*, years old.

**Table 4:** Margin of exposure (MoE) calculated for Cr in daily dose for each HMP with *Mentha x piperita* L., folium (A–J), depending on age and body weight for each specific population group (ng/kg bw/day)

Specific population groups. age	Approximate body weight (kg)	HMP with *Mentha x piperita* L.. folium									
		A	B	C	D	E	F	G	H	I	J
Children 3–6 y/o	15–23	2.85E + 07–4.36E + 07	2.03E + 07–3.11E + 07	1.08E + 08–1.66E + 08	7.61E + 07–1.17E + 08	3.79E + 07–5.81E + 07	2.00E + 07–3.07E + 07	1.03E + 08–1.58E + 08	3.69E + 07–5.66E + 07	2.82E + 07–4.32E + 07	7.06E + 07–1.08E + 08
Children 6–12 y/o	23 – 46	1.94E + 07–3.88E + 07	1.38E + 07–2.76E + 07	7.36E + 07–1.47E + 08	5.19E + 07–1.04E + 08	2.58E + 07–5.16E + 07	1.36E + 07–2.73E + 07	7.04E + 07–1.41E + 08	2.51E + 07–5.03E + 07	1.92E + 07–3.84E + 07	4.81E + 07–9.62E + 07
Adults	60	3.79E + 07	2.70E + 07	1.44E + 08	1.02E + 08	5.05E + 07	2.67E + 07	1.38E + 08	4.92E + 07	3.75E + 07	9.42E + 07

*y/o,* years old.

## Conclusion

Obtained raw results shows that impurities of total Cr impurities were present in all investigated HMP with *Mentha x piperita* L., folium (A–J) available in Polish pharmacies but at a relatively low level; in the range: 0.39–2.14 µg/L. The results obtained results are not coherent which explains the differences in composition and potentially different sources of raw material from manufacturers. We applied triple strategy based on regulatory purpose (WHO and EMA requirements) and ICH Q3D (R1) (guideline for elemental impurities) which confirms that all analyzed products meet the requirements (all results below 1100 µg Cr/g [10]). Furthermore, the obtained results show that the estimated maximum daily exposure of Cr (ng/day) is variable among analyzed samples (0.521–3.792 ng/day), but at a relatively low level (< 4.0 ng/day).The final step confirms the safety of the pharmaceuticals analyzed, because the comparison of the estimated results with the oral PDE value for Cr in the final drugs suggested by the ICH Q3D guideline (10,700 µg/day) show that all the products analyzed are below this value. The third approach based on margin of exposure (MoE) for children and adults also confirms the safety of all the products analyzed with *Mentha x piperita* L. (in all cases MoE >  > 10 000). It should be emphasized that this approach is very universal and powerful “toxicological tool” that can be applied to impurities that are both genotoxic and carcinogenic, regardless of their origin. Because two MoAs for Cr administered orally can occur and contribute to the genotoxic effects of Cr(VI), this approach was desired to fulfill modern TRA of total chromium impurities in the *Menthae piperitae tinctura* available in Polish pharmacies.

Further studies with application of the HPLC-ICP-MS technique would be appropriate to check the content of Cr(III) and Cr(VI), but in this work it is not necessary for regulatory purposes.

## Supplementary Information

Below is the link to the electronic supplementary material.

Supplementary file1 (DOCX 15 KB)

Supplementary file2 (DOCX 31 KB)

## Data Availability

All data generated or analyzed during this study are included in this published article and its supplementary information file.

## Abbreviations

AAS: Atomic absorption spectrometry technique
EMA: European Medicine Agency
EI: Elemental impurities
ICH Q3D: International Council for Harmonization of Technical Requirements for Pharmaceuticals for Human Use
MoE: Margin of exposure
PDE: Permitted daily exposure
THMP: Traditional herbal medicinal product
TRA: Toxicological risk assessment

## References

[1] Shariatifar N, Rezaei M, Alizadeh Sani M, Alimohammadi M, Arabameri M (2020). Assessment of rice marketed in Iran with emphasis on toxic and essential elements; effect of different cooking methods. Biol Trace Elem Res.

[2] Karami H, Shariatifar N, Nazmara S, Moazzen M, Mahmoodi B, Mousavi Khaneghah A (2021). The concentration and probabilistic health risk of potentially toxic elements (PTEs) in edible mushrooms (wild and cultivated) samples collected from different cities of Iran. Biol Trace Elem Res.

[3] Kleensang A, Maertens A, Rosenberg M, Fitzpatrick S, Lamb J, Auerbach S, Hartung T (2014). t4 workshop report pathways of toxicity. ALTEX.

[4] Torres S, Boetzel R, Gatimu E, Gomes DZ, King F, Kocks G, Teasdale A (2022). ICH Q3D drug product elemental risk assessment: the use of an elemental impurities excipients database. J Pharm Sci.

[5] Chatzopoulou M, Madden KS, Bromhead LJ, Greaves C, Cogswell TJ, Da Silva PS, Wynne GM (2022). Pilot study to quantify palladium impurities in lead-like compounds following commonly used purification techniques. ACS Med Chem Lett.

[6] Jurowski K, Fołta M, Tatar B, Krośniak M (2022). The level of cadmium impurities in traditional herbal medicinal products with *Plantago**lanceolata* L., folium (Ribwort plantain leaves) available in Polish pharmacies - comprehensive toxicological risk assessment including regulatory point of view and ICH Q3D elemental impurities guideline. Biol Trace Elem Res.

[7] Jurowski K, Krośniak M (2022). The toxicological assessment of content and exposure of heavy metals (Pb and Cd) in traditional herbal medicinal products with marshmallow root (Althaea officinalis L. radix) from Polish pharmacies. Toxics.

[8] Jurowski K, Mirosław K (2022). The human health risk assessment of heavy metals impurities (Cd and Pb) in herbal medicinal products as Menthae piperitae tinctura (Mentha × piperita L. folium) available in pharmacies from Poland. Toxics.

[9] Jurowski K, Fołta M, Tatar B, Krośniak M (2022). The comprehensive toxicological assessment of total chromium impurities in traditional herbal medicinal product with Thymi herba (Thymus vulgaris L. and Thymus zygis L) available in pharmacies in Poland. Short Communication..

[10] ICH guideline Q3D (R1) on elemental impurities. 28 March 2019EMA/CHMP/ ICH/353369/2013 Committee for human medicinal products.Available 12.05.2022 at https://www.ema.europa.eu/en/ich-q3d-elementalimpurities. Accessed 12 May 2022

[11] Knotek K, Verner V, Chaloupkova P, Kokoska L (2012). Prevalence and use of herbal products in the Czech Republic: over-the-counter survey among adult pharmacies clients. Compl Ther Med.

[12] Vincent JB (2017). New evidence against chromium as an essential trace element. J Nutr.

[13] Vincent JB, Brown S (2019) Introduction: a history of chromium studies (1955–2007). In: The Nutritional Biochemistry of Chromium (III), 1–58, Elsevier.

[14] EFSA Scientific Committee (2012). Statement on the applicability of the Margin of Exposure approach for the safety assessment of impurities which are both genotoxic and carcinogenic in substances added to food/feed. EFSA J.

[15] Rovida C, Asakura S, Daneshian M, Hofman-Huether H, Leist M, Meunier L, Hartung T (2015). Toxicity testing in the 21^st^ century beyond environmental chemicals. Altex.

[16] Zhitkovich A (2011). Chromium in drinking water: sources, metabolism, and cancer risks. Chem Res Tox.

[17] Bagchi D, Stohs SJ, Downs BW, Bagchi M, Preuss HG (2002). Cytotoxicity and oxidative mechanisms of different forms of chromium. Toxicology.

[18] Thompson CM, Proctor DM, Haws LC, Hébert CD, Grimes SD, Shertzer HG, Harris MA (2011). Investigation of the mode of action underlying the tumorigenic response induced in B6C3F1 mice exposed orally to hexavalent chromium. Tox Sci.

[19] EFSA Panel on Contaminants in the Food Chain (CONTAM) (2014). Scientific Opinion on the risks to public health related to the presence of chromium in food and drinking water. EFSA Journal.

[20] World Health Organization (2006) WHO guidelines for assessing quality of herbal medicines with reference to contaminants and residues, World Health Organization, Geneva, Switzerland. Available 12.05.2022 at https://apps.who.int/iris/handle/ 10665/43510 Accessed 12 May 2022

[21] National Toxicology Program. Technical report on the toxicology and carcinogenesis studies of chromium picolinate monohydrate (CAS NO. 27882–76–4) in F344/N rats and B6C3F1 mice (feed studies). National Toxicology Program, Public Health Service ,U.S. Department of Health and Human Services. 2010; NTPTR556. 20725156

[22] de Onis M, Onyango AW, Borghi E, Siyam A, Nishida C, Siekmann J (2007). Development of a WHO growth reference for school-aged children and adolescents. Bull World Health Organ.

