# Supplementary material for: The Toxicological Risk Assessment (TRA) of Total Chromium Impurities in Menthae piperitae tinctura (Mentha x piperita L., folium) Available in Polish Pharmacies Including Regulatory Approaches with Special Emphasis of Cr Speciation and Genotoxicity
Source: Biol Trace Elem Res. 2022 Jul 28;201(6):3060–8. doi: 10.1007/s12011-022-03367-4 (PMC10073164; doi:10.1007/s12011-022-03367-4)
Supplement: Supplementary file 1 — Supplementary file1 (DOCX 15 KB) [file 12011_2022_3367_MOESM1_ESM.docx]

**Supplementary materials 1 (SM1)**

The special time-temperature program for Cr determination was applied – table S1. The sample volume was 40 μL, and for signal evaluation, integrated absorbance (peak area) was applied.

**Table S1.** The special time-temperature program for Cr determination.

| Condition(s) | Value |
| --- | --- |
|  |  |
| Step 1, °C | 110 |
| Ramp/Hold, s | 5 / 15 |
| Step 2, °C | 180 |
| Ramp/Hold, s | 30/10 |
| Step 3, °C | 450 |
| Ramp / Hold, s | 1/5 |
| Step 4, °C | 500 |
| Ramp/Hold, s | 5/5 |
| Step 5, °C | 1500 |
| Ramp/Hold, s | 15/30 |
| Step 6, °C | 2450 |
| Ramp/Hold, s | 0/5 |
| Step 7, °C | 2500 |
| Ramp/Hold, s | ½ |
| L’vov platform | Yes |
| Integration time, s | 4 |
| Injected: sample volume, µL | 40.0 |

The analytical calibration strategy was applied including working solutions of Cr (5.0; 10; 50.0, 100.0 and 150.0 μg/L) prepared from the stock solutions of 1000 μg/mL (chromium(III) nitrate; CertiPUR®) using ultrapure demineralized water in 0.5 mol/L nitric acid. The calibration function indicated an acceptable correlation coefficient (R = 0.9989). Hence there was good linearity of instrumental response with metal concentrations.

For quality control, the certified reference material was applied (lichen; BCR-482 IRMM, Belgium); the certified value for Cr was 0.134 mg/kg, and the measured value was 0.137 mg/kg. Hence the recovery was 97.81%. The LOD was 1.65 μg/L, and LOQ was 4.95 μg/L.

All data were analyzed using statistical software Origin 2021 Pro, the Ultimate Software for Graphing and Analysis (OriginLab Corporation, One Roundhouse Plaza, Suite 303, Northampton, MA 01060, USA) licensed by the Jagiellonian University in Krakow. The resultant data of five independent replicates (five replicate samples from one bottle of each product) were expressed as the mean ± standard deviation.
