# Supplementary material for: The Toxicological Risk Assessment (TRA) of Total Chromium Impurities in Menthae piperitae tinctura (Mentha x piperita L., folium) Available in Polish Pharmacies Including Regulatory Approaches with Special Emphasis of Cr Speciation and Genotoxicity
Source: Biol Trace Elem Res. 2022 Jul 28;201(6):3060–8. doi: 10.1007/s12011-022-03367-4 (PMC10073164; doi:10.1007/s12011-022-03367-4)
Supplement: Supplementary file 2 — Supplementary file2 (DOCX 31 KB) [file 12011_2022_3367_MOESM2_ESM.docx]

**Supplementary materials 2 (SM2)**

Raw results of total Cr content in analysed HMP with *Mentha x piperita* L., folium (A – J) are presented in Table S2.

**Table S2.** Raw results of total Cr content in analysed HMP with *Mentha x piperita* L., folium (A – J).

| Sample | | total Cr level, µg/kg | |
| --- | --- | --- | --- |
| No. | Code | Mean | SD |
| 1. | A | 1.504 | 0.09 |
| 2. | B | 2.112 | 0.08 |
| 3. | C | 0.396 | 0.03 |
| 4. | D | 0.562 | 0.07 |
| 5. | E | 1.131 | 0.08 |
| 6. | F | 2.141 | 0.03 |
| 7. | G | 0.414 | 0.07 |
| 8. | H | 1.161 | 0.08 |
| 9. | I | 1.521 | 0.09 |
| 10. | J | 0.606 | 0.08 |

The descriptive statistics of Cr content in analysed HMP with *Mentha x piperita* L., folium
(A – J) is presented in Table S3.

**Table S3**. The descriptive statistics of Cr content in analysed HMP with *Mentha x piperita* L., folium (A – J)

| parameter | Minimum, μg/L | Maximum, μg/L | Mean, μg/L | Skewness | Kurtosis |
| --- | --- | --- | --- | --- | --- |
| value | 0.39 | 2.14 | 1.15 | 1.48 | 2.63 |

*SD* standard deviation
